# Rational Design of Fluorinated Phthalonitrile/Hollow Glass Microsphere Composite with Low Dielectric Constant and Excellent Heat Resistance for Microelectronic Packaging

**DOI:** 10.3390/nano12223973

**Published:** 2022-11-11

**Authors:** Minjie Wu, Wenshuang Han, Chun Zhang, Shuo Zhang, Xinyang Zhang, Xinggang Chen, Kimiyoshi Naito, Xiaoyan Yu, Qingxin Zhang

**Affiliations:** 1 Hebei Key Laboratory of Functional Polymers, School of Chemical Engineering and Technology, Hebei University of Technology, Tianjin 300401, China; 2 School of Materials Science and Engineering, North China University of Science and Technology, Tangshan 063210, China; 3 National Institute for Materials Science (NIMS), Hybrid Materials Unit, Composite Materials Group, 1-2-1 Sengen, Tsukuba 305-0047, Japan; 4 Tianjin Key Laboratory of Materials Laminating Fabrication and Interface Control Technology, Hebei University of Technology, Tianjin 300401, China

**Keywords:** fluorinated phthalonitrile, hollow glass microsphere, low dielectric constant, thermal mechanical properties, electronic packaging

## Abstract

High-performance composites with a resin matrix are urgently required for electronic packaging due to their low dielectric constant, outstanding high temperature resistance, excellent corrosion resistance, light weight and easy molding. In this work, hollow-glass-microsphere (HGM)-filled fluorinated-phthalonitrile (PBDP) composites, with filler contents ranging from 0 to 35.0 vol.%, were prepared in order to modify the dielectric properties of the phthalonitrile. Scanning electron microscopy (SEM) observations indicate that the modified HGM particles were uniformly dispersed in the matrix. The PBDP/27.5HGM-NH_2_ composite demonstrates a low dielectric constant of 1.85 at 12 GHz. The 5% thermogravimetric temperature (*T*_5_) of composites with silanized HGM filler (481–486 °C) is higher than the minimum packaging-material requirements (450 °C). In addition, the heat-resistance index (*T*_HRI_) of PBDP/HGM-NH_2_ composites reached as high as 268 °C. the storage modulus of PBDP/HGM-NH_2_ composites were significantly increased to 1283 MPa at 400 °C, an increase by 50%, in comparison to that of PBDP phthalonitrile resin (857 MPa). The excellent dielectric and thermal properties of the present composites may pave a way for comprehensive applications in electronic packaging and thermal management for energy systems.

## 1. Introduction

With the continuous high integration and miniaturization of electronic devices, the delay, crosstalk and power consumption caused by the resistance and capacitance of metal wires and dielectric layers have become the key factors limiting devices [1,2]. Reducing the dielectric constant of the packaging material is helpful to improve the signal transmission speed, and reduce the signal delay and the signal loss [3,4]. Therefore, the development of low dielectric constant insulating materials in electronic packaging and substrate applications is one of the most attractive topics.

Composites play an important role in the electronic and electrical field, including metal-matrix, ceramic-matrix and resin-matrix composites. Metal-matrix and ceramic-matrix composites have attracted the attention of many researchers for their good conductivity and thermal conductivity. Resin-matrix composites are attracting more and more interest in the field of electronic packaging because of their good insulation, processability, corrosion resistance, etc. A low dielectric constant is one of the most crucial design criteria in selecting suitable resin-matrix composites [5,6,7]. The study of low-dielectric-constant materials is closely related to polymer resins. Due to their inherent low dielectric constant, low water absorption, excellent processability, light weight and preferable dimensional stability, polymeric materials have attracted extensive attention in both academia and industry [8]. At present, a large number of polymers with low dielectric constants have been proposed as insulating materials, such as polybenzoxazine [9,10], poly(imide)s [11,12], poly(aryl ether)s [1,13], poly(ether ketone)s, cyanate ester [14], polysiloxanes and phthalonitrile [15,16]. Generally, insulating resin materials with a low dielectric constant can be obtained by reducing the dipole strength or density within the resins [8,17,18]. For instance, Ya. L. Kobzar et al. [19] described the design and synthesis of novel corefluorinated polybenzoxazines with low polarizability which showed a dielectric constant of 2.61. Ihor Tkachenko et al. [13] prepared fluorinated poly(arylene ether)/silica cross-linked materials (FPAE/SiO_1.5_) with high hydrophobic properties (water contact angles above 102°), low dielectric constants and losses at room temperature. In addition, B. L. Zhu et al. [20] incorporated the surface of S60HS HGM with KH570 into LDPE. The dielectric constant of the material decreased from 2.34 to 2.25, when the HGM addition content was 50 vol.%. Joseph et al. [21] added hollow polyhedral oligomeric silsesquioxane (POSS) into polystyrene (PS), which effectively reduced the density of the composite, so that the low dielectric constant value of the composite decreased to 1.95. At the same time, the thermal stability of the composite was enhanced and the coefficient of thermal expansion was reduced. In fact, the introduction of a hollow structure into polymer materials is a more realistic method of achieving a low dielectric constant without the loss of material thermal stability.

On the other hand, some electronic devices and composites work at high ambient temperatures. Hence, it is natural to choose a high-performance polymer with excellent heat resistance as a dielectric material matrix. Phthalonitrile (PN), characterized by solvent-free addition polymerization, is one class of very promising electronic-packaging materials because of their excellent thermal stability and low dielectric constant. Moreover, PN is endowed with good dimensional stability, preferable high-temperature mechanical properties, excellent heat and humidity resistance, and low water adsorption, etc. [22,23,24,25]. It is a promising candidate in aerospace, electronics, insulation, adhesives and other fields. However, PN resin needs to be modified to meet the dielectric properties of electronic-packaging materials. In previous reports [16,26], researchers tried to introduce fluorine atoms into PN resin to reduce the dielectric constant of resin, and achieved certain results. Herein, hollow glass microspheres (HGM) were introduced into the fluorophthalonitrile (PBDP) matrix prepared by our research group [26], to obtain a PN composite with low dielectric constant, excellent thermodynamic mechanical properties and high heat resistance. HGM consist of outer stiff glass and inner inert gas, which results in some superior characteristics such as a low dielectric constant, light weight and excellent thermal insulation and sound insulation. It has been reported that HGM can improve the dielectric properties of polymers [4,20,27].

To the best of our knowledge, there are few reports on the preparation of low-dielectric-constant and high-heat-resistance composites by introducing hollow HGM into PN resin. In this work, the HGM (S60HS) was used as filler and the fluorophthalonitrile PBDP with low dielectric-constant values was chosen as the matrix. Firstly, (3-Aminopropyl)triethoxysilane (APTES) was applied to modify HGM and, thus, improve the compatibility between matrix and filler. Next, as-modified HGM (5, 10, 15, 20 wt.%) was incorporated into the PBDP matrix to prepare the PBDP/HGM composite using the melting–casting method. The resulting PBDP/HGM composites were provided with a low dielectric constant and preferable thermal-dynamic mechanical properties, which are expected to be used in areas of electronic-packaging material and aerospace.

## 2. Materials and Methods

### 2.1. Materials

HGM (trade name: S60HS, ρ_HGM_: 0.60 g/cm^3^) were purchased from 3M Co., Ltd. (St. Paul, MN, USA). The chemical composition of HGM mainly includes silicon dioxide (SiO_2_), calcium oxide (CaO), boron oxide (B_2_O_3_) and sodium oxide (Na_2_O). The scanning electron microscope (SEM) micrograph of the original HGM is shown in Figure S1. (3-Aminopropyl)triethoxysilane (APTES) were obtained from McLean Co., Ltd. (Shanghai, China). Xylene was produced and supplied by Guangfu Fine Chemical Research Institution (Tianjin, China). Sodium hydroxide (NaOH) was provided by Kemeiou Chemical Reagent Co., Ltd. (Tianjin, China). In addition, 4-(aminophenoxy)phthalonitrile (APPH) [28] and 4,4′-bis(*p*-6+erfluoro-phenol-(bis(*p*-phenol)propane-2,2-diyl)-*p*-oxy-diphthalonitrile) (PBDP) [26] were prepared by our laboratory. The density of PBDP was 1.29 g/cm^3^.

### 2.2. Surface Modification of HGM with APTES (HGM-NH_2_)

**Figure 1:**
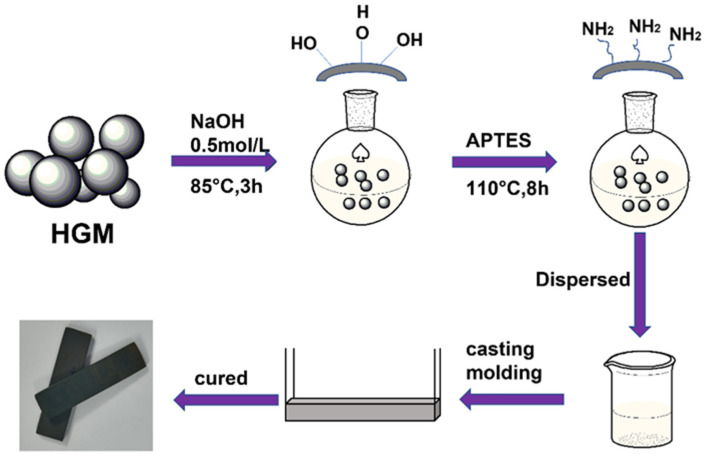
The preparation procedures of modified particles and PBDP/HGM-NH_2_ composites.

The preparation procedures of modified particles are schematically represented in Figure 1. First, 1 g of HGM was ultrasonically dispersed into 100 mL as-prepared NaOH solution (0.5 mol/L) and magnetically stirred at 85 °C for 3 h. The process was to activate the surface of HGM and increase the number of hydroxyl groups on the surface. The mixture was filtered and the particles were washed to neutral with deionized water. The collected resultant was dried for 12 h at 60 °C and expressed as HGM-OH for further use.

To prepare the silanized particles, 10 g HGM-OH particles were first added into 500 mL xylene and the mixture was ultrasonically dispersed for 30 min and then magnetically stirred at 40 °C for 1 h. After that, 1.5 g APTES was added dropwise into the mixture solution, stirred for 7 h at 110 °C, then filtered and washed with EtOH to wash the unhydrolyzed APTES. Finally, the product was dried at 60 °C for 24 h, to give HGM-NH_2_.

### 2.3. Preparation of Phthalonitrile Monomer as Matrix of Composite Materials

**Scheme 1:**
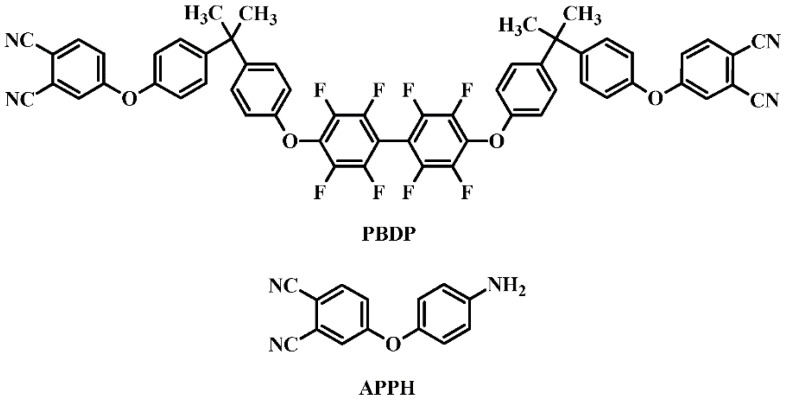
The structural formula of PBDP and APPH.

The structural formula of PBDP and APPH are shown in Scheme 1. Synthesis, characterization and comprehensive properties of PNs resin have been presented in our previously reported paper [26,28]. PBDP resin, characterized by outstanding thermal stability, low dielectric constant and water absorption, is a good choice for matrix because of its low melting point (96 °C), wide processing window (96–262 °C) and low melting viscosity (<2 Pa·s). In the absence of active hydrogen provided by curing agent, APPH released itself to provide active hydrogen atom during heating, which initiates the thermal polymerization of phthalonitrile group. The APPH resin can be used as curing agent in thermosetting reaction.

### 2.4. Preparation of the PBDP/xHGM Composites

The volume percent of HGM-NH_2_ in the composites was determined using the following Equation (1):

(1) $$V_{f}=\frac{W_{f}}{W_{f}+\left(1-W_{f}\right)\frac{\rho_{f}}{\rho_{r}}}$$

where *W_f_* is the HGM-NH_2_ weight fraction (5, 10, 15 and 20 wt.%) of the composites; *ρ_f_* and *ρ_r_* are the densities of the HGM and PBDP resin, respectively.

Figure 1 shows the preparation process of the PBDP/HGM-NH_2_ composites. The composites were prepared as follows: HGM-NH_2_, PBDP, and APPH were dispersed by water-phase mixing and ultrasonic dispersion at room temperature for 30 min, and the mixture was filtered and dried for standby. The PBDP/xHGM-NH_2_ composites were produced using a casting method, where the mass ratio of PBDP and APPH was 9:1, and x represents volume fraction of HGM in the polymer, carrying values of 10.2, 19.3, 27.5, and 35.0 vol.%. The compound was melted at 200 °C in a vacuum oven to remove bubbles. The smooth gelatinization compound was cured and post-cured following the process of 200 °C × 2 h, 230 °C × 2 h, 260 °C × 4 h, 290 °C × 4 h, 320 °C × 4 h, 350 °C × 4 h, and 380 °C × 6 h, successively, to obtain cured PBDP/HGM-NH_2_ composites.

### 2.5. Instrumentation

A Fourier transform infrared (FT-IR) spectrometer (Shimadzu IRAffinity-1S) (Tianjin, China) was used to investigate the surface functional groups of HGM before and after modification. The wavenumber was ranged from 4000 to 400 cm^−1^ using the KBr slice as scanning background. Its resolution was 4 cm^−1^ and the scan time was 32. The dry KBr pellets were mixed with the powders of the samples by grinding for homogenization. The mixed powder was then pressed into a transparent and thin slice. These thin slices were used for the FTIR spectral measurements. The KBr pellets and HGMs were fully dried in the drying oven before testing. In addition, the background subtracted from the sample spectra.

A TA Instruments Q600 thermogravimetric analyzer (Waltham, MA, USA) was employed to measure the thermogravimetric mass before and after HGM modification in nitrogen atmosphere (about 8 mg) from room temperature to 800 °C under the heating rate of 10 °C min^−1^. The thermal stability of PBDP/xHGM-NH_2_ composites was determined by thermogravimetric analysis (TGA) in air (O_2_ + N_2_) atmosphere (about 8 mg) with a heating rate of 10 °C min^−1^ from 30 °C to 1000 °C. The gas flow rate was 100 mL min^−1^ in all cases. Microstructural morphologies and energy dispersive spectrometer (EDS) of PBDP/xHGM-NH_2_ composites were characterized by scanning electron microscope (SEM; Nova Nano SEM450; FEI (Hillsborough, OR, USA)) operating at 20 kV. Samples were sprayed with gold coating prior to SEM analyses.

Differential scanning calorimetric (DSC) analysis was collected on a TA Q20 (Waltham, MA, USA) at a heating rate of 10 °C min^−1^ with a nitrogen flow rate of 80 mL/min.

Relaxation dynamics and elastic properties of the PBDP/xHGM-NH_2_ composites were estimated by dynamic mechanical analysis (DMA), which was performed using a TA Instruments Q800 dynamic mechanical spectrometer (Waltham, MA, USA) at 1 Hz over the temperature range from 30 to 400 °C, at a heating rate of 5 °C min^−1^. The working part of test samples was of ca. 40 × 10 × 2 mm^3^.

Dielectric constant and dielectric loss of PBDP/xHGM-NH_2_ composites were carried out using an Aligent PNA-N5244A Network Analyzer (Palo Alto, CA, USA). The experiment was performed at room temperature in the frequency range from 8.2 to 12.4 GHz.

## 3. Results and Discussion

### 3.1. Characterization of HGM before and after Functionalization

**Figure 2:**
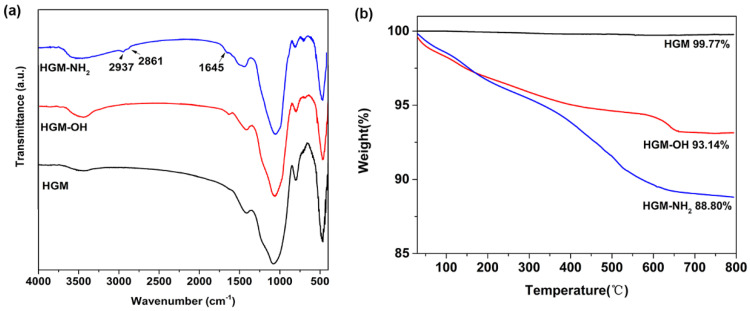
FT-IR spectra (**a**) and TGA curves (**b**) of HGM before and after modification.

In order to study the surface groups of HGMs, a series of characterizations were carried out. As shown in the Figure 2a, in all the FT-IR spectra of HGM, HGM-OH and HGM-NH_2_, there are typical absorption peaks at ~1078, ~798 and ~464 cm^−1^, corresponding to the stretching vibration of Si–O–Si bonds. HGM and HGM-OH samples have a wide peak in the range of 3400~3200 cm^−1^, which belongs to the –OH vibration peak. Obviously, –OH on HGM after NaOH activation is more abundant. However, the absorption peak of Si–OH at about 960 cm^−1^ may be covered by the strong absorption peak of Si-O (1068 cm^−1^) of HGM. After the modification with APTES, the HGM-NH_2_ shows new absorption peaks at 2937 cm^−1^ and 2861 cm^−1^, which are the asymmetric and symmetric stretching of the –CH_2_– bond. In addition, the spectrum of HGM-NH_2_ shows a peak at 1645 cm^−1^ corresponding to the –NH_2_ deformation vibration of primary amine groups [10,29]. The Si–O–Si absorption peak generated by the APTES modification reaction may be covered by the original Si–O absorption peak of HGM. It proves that an APTES molecule was grafted onto HGM-OH successfully. Meanwhile, the TGA curves of HGMs in Figure 2b give further evidence of successful surface modification. In the temperature range of 30–800 °C, it can be seen from Figure 2b that pure HGM has no significant weight loss, which proves its excellent thermal stability. The TGA curves of HGM-OH and HGM-NH_2_ all presented less weight loss before 300 °C, which is due to the desorption of physically adsorbed water and decomposition of unstable oxygen-containing functional groups on the surface of particles. Compared with HGM-OH, HGM-NH_2_ modified by APTES showed a weight loss of 4.34 wt.% within the same temperature range. The main mass loss areas of HGM-NH_2_ happened at ~600 °C, caused by the silica silane coupling agent. This result also supports the successful modification of HGM by APTES [30]. In addition, the EDS spectra of HGM and HGM-NH_2_ are shown in Figure S2 (the corresponding data are listed in Table S1).

### 3.2. Cure Studies on PBDP/HGM-NH_2_ Blends

**Figure 3:**
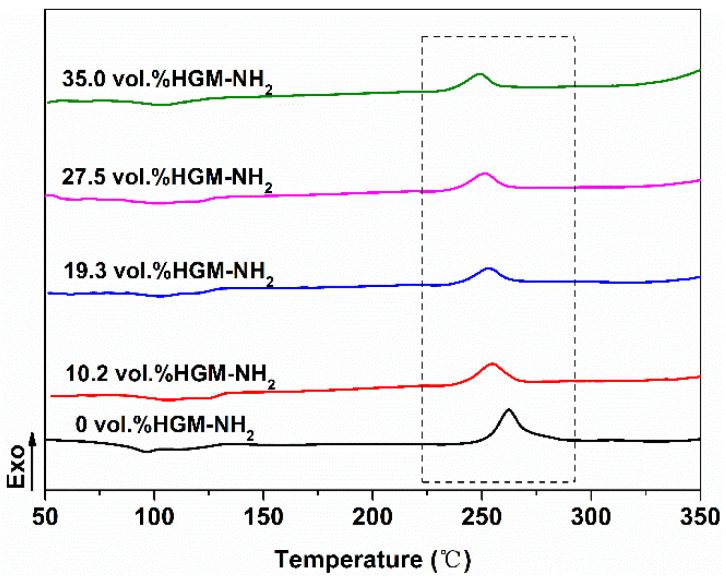
DSC curves of PBDP/HGM-NH_2_ blends with different weight percentage of HGM-NH_2_ (0~35.0 vol.%).

The DSC were used to investigate the curing reactions of PBDP/HGM-NH_2_ blends; results are presented in Figure 3. PBDP/HGM-NH_2_ blends with various contents of HGM-NH_2_ all showed an exothermic peak corresponding to the thermal polymerization of nitrile groups with APPH as curing agent. The exothermic peaks in the dash box were the curing peak of the PBDP/xHGM-NH_2_ system. The curing exothermic peak temperature of composites (248.5~254.3 °C) were lower than that of the PBDP/APPH blend (257.5 °C). With an increase in HGM-NH_2_ content, the peaks shift to lower temperatures (254.3 °C, 252.9 °C, 251.3 °C and 248.5 °C for 10.2, 19.3, 27.5 and 35.0 vol.%, respectively). This can be attributed to the amino and hydroxyl groups on HGM surface promote the curing reaction of phthalonitrile.

### 3.3. Microstructure of the Composites

**Figure 4:**
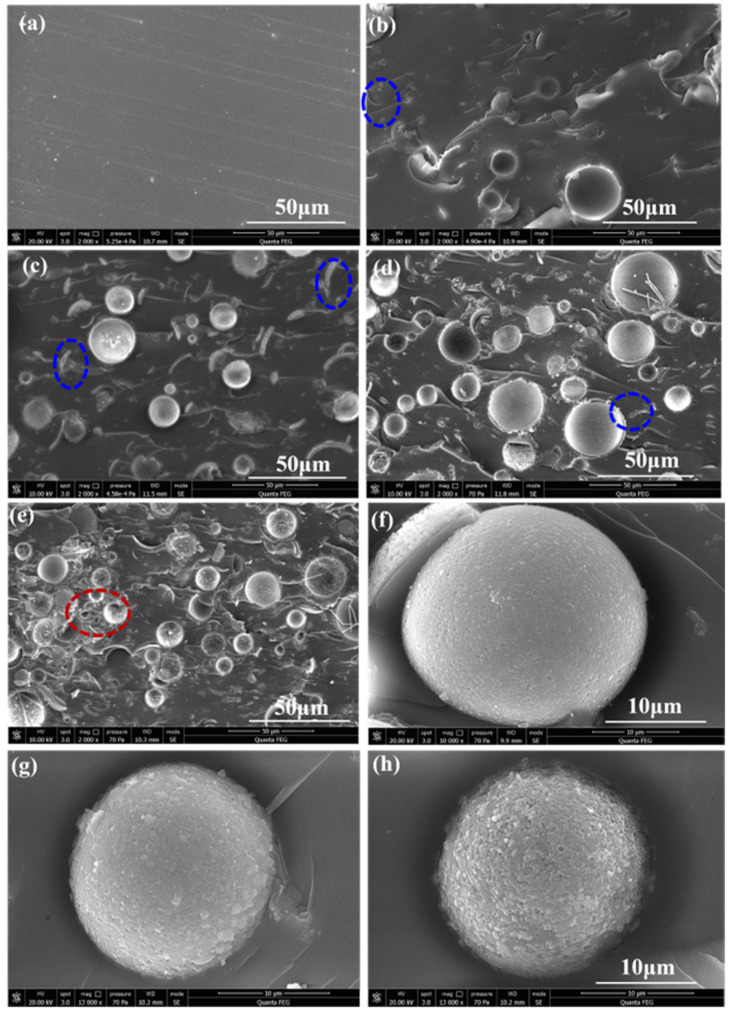
SEM images of the PBDP phthalonitrile resin and composites fracture surface: (**a**) PBDP phthalonitrile resin, (**b**) PBDP/10.2HGM-NH_2_, (**c**) PBDP/19.3HGM-NH_2_, (**d**) PBDP/27.5HGM-NH_2_, (**e**) PBDP/35.0HGM-NH_2_, (**f**) PBDP/19.2HGM-NH_2_, (**g**) PBDP/27.5HGM-NH_2_, (**h**) PBDP/37.5HGM. (The blue circle indicates broken HGM-NH_2_ particles, and the red circle indicates slight agglomeration phenomenon).

Usually, the cryofractured surfaces of composites are observed by SEM with the aim of establishing the microstructure of the composites and the dispersion degree of the fillers in the matrix [14,21,31,32]. Figure 4 shows SEM micrographs of the fracture surface of PBDP–resin and PBDP/xHGM-NH_2_ composites.

**Figure 5:**
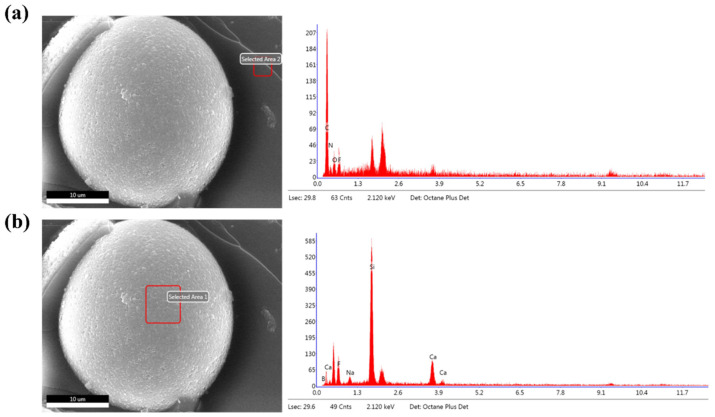
EDS point scanning in composite: (**a**) PBDP resin, (**b**) HGM-NH_2_.

Figure 4a displays SEM micrographs of the fracture surface of the PBDP phthalonitrile matrix, and the fracture surface is flat and smooth. Figure 4b–e are SEM micrographs of the fracture surface of PBDP/xHGM-NH_2_ composites. The cross-section morphology of the composite shows that there is no obvious particle agglomeration on the fracture surface of the composites, indicating that silanized HGM particles are well-dispersed in PBDP phthalonitrile resin. Figure 4f–h are SEM micrographs of the fracture surface of PBDP/19.3HGM-NH_2_, PBDP/27.5HGM-NH_2_ and PBDP/27.5HGM composites at higher magnifications, respectively. The HGM-NH_2_ are found to be tightly contacted with PBDP. However, the gap between HGM and PBDP are observed in pristine filler-filled composites. This is because the coupling agent connects the HGM-NH_2_ with phthalonitrile resin by chemical force. One end of the coupling agent has an amino group which is compatible with phthalonitrile resin and can participate in the curing reaction, and the other end of the coupling agent has a secondary bond force with the HGM, which improves the interfacial compatibility and the interfacial adhesion effectively [20,33]. Figure 5 shows the EDS point scanning and results of PBDP and HGM-NH_2_ position images in the PBDP/30HGM-NH_2_ composite. In the HGM-NH_2_ position, in addition to the signal of the characteristic elements Si, B, Ca and Na of HGM, there is also the signal of element F in PBDP resin. In a certain sense, PBDP resin is coated on the surface of HGM-NH_2_. However, a small number of broken HGM-NH_2_ particles of the composites can be observed in the Figure 4, marked by blue circles in the figure. When the addition of HGM-NH_2_ at 35.0 vol.%, the slight agglomeration phenomenon appears in the composite, which is marked by a red circle in the figure. The imperfection and agglomeration of HGM-NH_2_ may have a negative effect on the comprehensive properties of the composites [31].

### 3.4. Dielectric Properties

**Figure 6:**
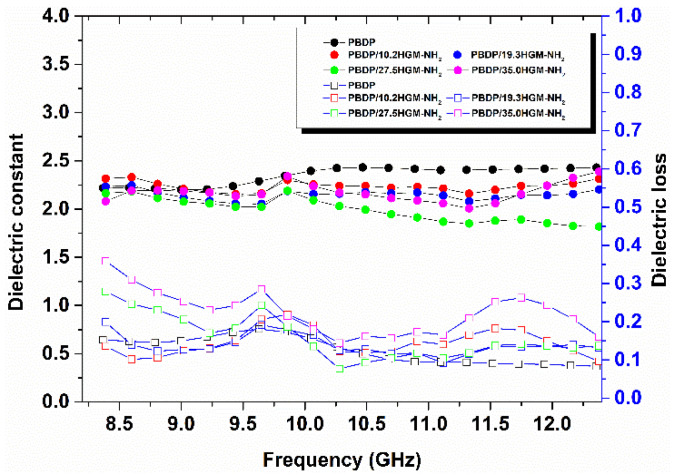
Dielectric properties as a function of frequency for neat, PBDP/HGM-NH_2_ composites.

The dielectric constant is a key parameter of packaging materials, which determines the signal propagation of electronic devices. Low-dielectric-constant packaging materials can effectively reduce dynamic power consumption and improve operation speed [34,35]. To evaluate the dielectric constant and dielectric loss of the PBDP/HGM-NH_2_ composites, dielectric parameters were measured using a Aligent PNA-N5244A Network Analyzer. Figure 6 presents the dielectric properties of the matrix and PBDP/HGM-NH_2_ composites in the frequency range of 8.2 GHz–12.4 GHz. The dielectric constant of PBDP/HGM-NH_2_ composites are lower than the PBDP phthalonitrile resin over the whole frequency range.

As mentioned above, insulating resin materials with a low dielectric constant can be obtained by reducing the dipole strength or density within the resins. Actually, the dielectric constant could be determined according to the Debye Equation (2) [8]:

(2) $$\frac{\varepsilon -1}{\varepsilon +2}=\frac{4\pi }{3}N\left(\alpha_{e}+\alpha_{d}+\frac{\mu^{2}}{3k_{b}T}\right)$$

where *ε*, *N*, *α_e_*, *α_d_*, *μ*, *k_b_* and *T* represent the dielectric constant, number of the density of dipoles, electric polarization, distortion polarization, orientation polarization related to the dipole moment, Boltzmann constant and temperature, respectively.

As can be seen in Figure 5, the dielectric constant of PBDP resin and PBDP/HGM-NH_2_ composites are ~2.43 and 2.31~1.85, respectively. The minimum dielectric constant of the PBDP resin with 27.5 vol.% HGM-NH_2_ decreased to 1.85, which was lower than that of the pure PBDP matrix (2.43). The greatly decreased dielectric constant is mainly ascribed to the introduction of pore structure in phthalonitrile materials. HGM is a kind of hollow particle, and its addition is conducive to reducing the number of the density of the dipoles (*N*) of phthalonitrile resin, thus reducing the dielectric constant of the material. In addition, air has the lowest dielectric constant of about 1. The air stored in the HGM pores is conducive to reducing the dielectric constant (*ε*) of the composites [1,20].

Moreover, as described in “3.3 Microstructure of the composites”, the modified HGM can be well dispersed in the matrix, being helpful to reduce the dielectric constant of the material. With the increase in HGM-NH_2_ content, the total amount of air in the matrix increases, and the dielectric constant of the composite is supposed to continue to decline. However, the experimental results show that the dielectric constant of the composite increases when the HGM-NH_2_ content is 35.0 vol.%. This may be due to the excessive amino group on the surface of HGM-NH_2_ particles, which is the polar group, leading to the increase in dipole polarization in the material [36,37]. Meanwhile, with the increase in filler content, agglomeration occurs to form large particles, which aggravates the polarization of the interface between the two phases, and the accumulation of electrons at the interface increases the macroscopic response of the material to the external electric field [31,38]. These factors counteract the influence of air, so the dielectric constant of the PBDP/35.0HGM-NH_2_ composite increases instead. For the present study, the dielectric constant values of composites (2.31~1.85) are smaller than those in previously reported results of HGM–filled epoxy composites (2.84–3.98) [27], TPHQPh/QF (~3.4), TBBPPh/QF (~3.5), TBRSPh/QF (~3.7) [39] and GO-filled PN (4.25–2.66) [31].

The introduction of inorganic fillers into an insulating resin usually results in high dielectric loss [40,41]. Figure 6 reveals the dielectric loss of both PBDP resin and PBDP/HGM materials is kept below 0.35. Compared with that of PBDP resin, the obtained dielectric loss of PBDP/HGM-NH_2_ composites is relatively higher, which may be caused by the following two reasons: the HGM possesses a relatively higher dielectric loss value in comparison to that of the matrix; or there is interface loss between HGM-NH_2_ and phthalonitrile resin [20].

From the overall observations, it can be concluded that PBDP/HGM-NH_2_ composites are showing lower dielectric constant and dielectric loss values, which make them applicable in electronics industries.

### 3.5. Thermal Properties

**Figure 7:**
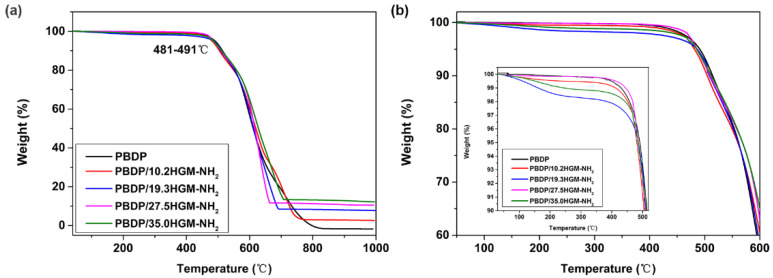
(**a**) TG curves of the neat PBDP and PBDP/HGM-NH_2_ composites, (**b**) extended view of TG curve with weight loss of 60~100% and enlarged view of with weight loss of 90~100%.

Thermal stability is one of the important properties of composite materials, which directly affects the service-life performance and application fields of materials. In addition, thermal stability is an important parameter of packaging materials, because the formation of metal interconnects on packaging materials is completed at high temperatures [42]. The thermal decomposition of PBDP/HGM-NH_2_ composites under an air atmosphere was investigated by TGA at the rate of 10 °C/min. TG curves of PBDP/HGM-NH_2_ composites are presented in Figure 7, and corresponding characteristic thermal data are summarized in Table 1.

(3) $$T_{HRI}=0.49*\left[T_{5}+0.6*(T_{30}-T_{5}\right]$$

As can be seen in Figure 7, the weight loss of PBDP/HGM-NH_2_ composites can hardly be detected before 400 °C in air. The 5% weight-loss temperature (*T*_5_) of the cured pure resin and PBDP/HGM-NH_2_ composites is around 490 °C, but the *T*_5_ of composites with silanized HGM filler (481–486 °C) is slightly lower than that of pure resin. This may be due to the presence of polar groups such as amino and hydroxyl groups on the surface of functionalized HGM particles, which have lower thermal decomposition ability compared with the PBDP [10]. It is even more surprising that the *T*_5_ of the PBDP/HGM-NH_2_ composites is higher than the minimum packaging-material requirements (450 °C) [34,44]. The heat-resistance index (*T*_HRI_) of PBDP/HGM-NH_2_ composites in air is 265.0–268.4 °C, which is similar to that of the resin matrix. On the whole, the PBDP/HGM-NH_2_ composite still maintains excellent thermal stability.

**Figure 8:**
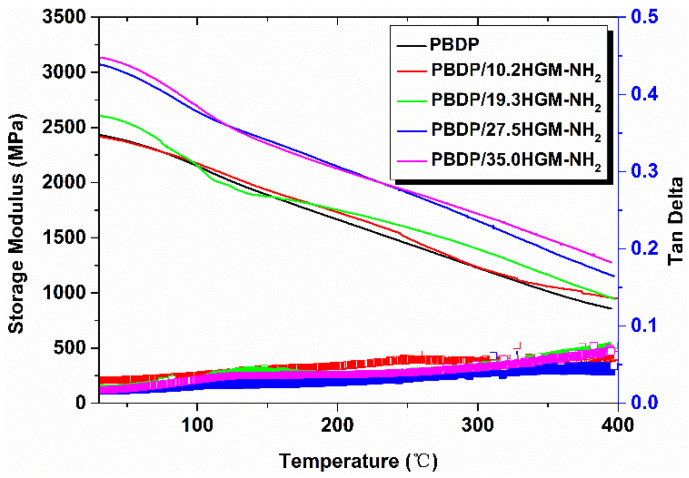
DMA curves of the neat PBDP, PBDP/HGM-NH_2_ composites.

Dynamic mechanical analysis (DMA) was performed to evaluate the mechanical behavior of the composites. As shown in Figure 8, all *tanδ* (T) plots have no obvious peak value, indicating that the glass transition temperature (*T*_g_) of the composites is higher than 400 °C, and the composites still maintain excellent heat resistance. Plots of the dynamic modulus versus (*T*) show that the storage modulus of PBDP/HGM-NH_2_ composites with 10.2%, 19.3%, 27.5% and 35.0 Vol.% HGM-NH_2_ at 30 °C is 2407, 2602, 3072 and 3135 MPa, respectively, and the storage modulus decreases slowly with temperature increases. At about 400 °C, the storage modulus of the composite with a 35.0 Vol.% HGM-NH_2_ filler increases to 130% of that of the pure resin. The reason is that the surface functionalization of HGM-NH_2_ can effectively increase the interfacial compatibility between filler and matrix. In addition, the HGM-NH_2_ filler restricts the motion of the polymer chain [45,46].

**Figure 9:**
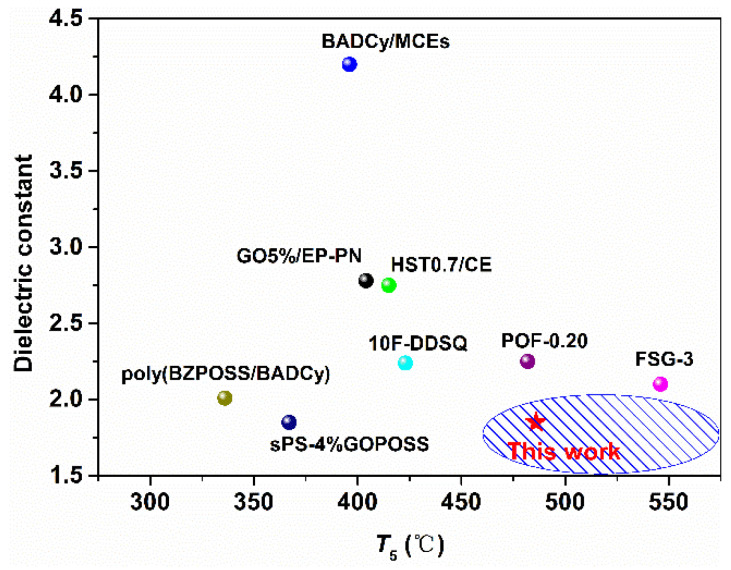
The dielectric constant for PBDP/HGM-NH_2_ composites in this work and other polymer composites reported in the literature vs. thermal decomposition temperature.

As shown in Figure 9, the dielectric constant of the PBDP/HGM-NH_2_ composites as compared with that of those reported composites [31,37,47,48,49,50,51,52] along with their *T*_5_s are listed herein. The results show that PBDP/HGM-NH_2_ composite has lower dielectric constant and excellent heat-resistance performance. It can be predicted that the PBDP/HGM-NH_2_ composite can be used as high-performance structural and functional materials, especially high-frequency electronic-packaging materials.

## 4. Conclusions

In summary, we developed a fluorinated phthalonitrile (PBDP) composite with outstanding comprehensive performance through blending with HGM-NH_2_. SEM images showed that HGM-NH_2_ particles were dispersed in matrix. The prepared HGM-NH_2_-filled composites have a low dielectric constant (2.31~1.85), meritorious thermal stability (*T*_5_ > 450 °C), excellent *T*_HRI_ as high as 268 °C and high *T*_g_ (>400 °C). The merits of the designed composites are suggested to originate from the excellent intrinsic properties of the PBDP matrix, and the uniform embedding of the modified HGM. These composites have application prospects in the microelectronics, aeronautic and military fields for their low dielectric constant, excellent thermal stability and remarkable thermal mechanical properties.

## Figures and Tables

**Table 1:** Thermal-characteristic data of the PBDP/HGM-NH_2_ composites.

Samples	*T*_5_ (°C)	*T*_10_ (°C)	*T*_30_ (°C)	*T*_HRI_ (°C)	*Y*_C_ (%)
PBDP	490.8	514.1	577.6	266.0	0
PBDP/10.2HGM-NH_2_	481.1	504.6	580.7	265.0	2.8
PBDP/19.3HGM-NH_2_	484.3	503.5	579.7	265.3	8.0
PBDP/27.5HGM-NH_2_	485.7	509.7	588.3	268.1	10.7
PBDP/35.0HGM-NH_2_	484.9	511.9	589.5	268.4	12.8

Note. The heat-resistance index of composites is calculated using Equation (3) [42,43]. *T*_5_, *T*_10_ and *T*_30_ are corresponding decomposition temperature of 5%, 10% and 30% weight loss, respectively; *Y*_C_ represents the carbon residue rate.

## Data Availability

Not applicable.

## Supplementary Materials

The following supporting information can be downloaded at: https://www.mdpi.com/article/10.3390/nano12223973/s1, Figure S1: SEM micrograph of the original HGM; Figure S2: EDS spectra of HGM and HGM-NH_2_; Table S1: HGM and HGM-NH_2_ atomic content determined by EDS spot scanning.
