# Supplementary material for: Rational Design of Fluorinated Phthalonitrile/Hollow Glass Microsphere Composite with Low Dielectric Constant and Excellent Heat Resistance for Microelectronic Packaging
Source: Nanomaterials (Basel). 2022 Nov 11;12(22):3973. doi: 10.3390/nano12223973 (PMC9698618; doi:10.3390/nano12223973)
Supplement: Supplementary file 1 [file nanomaterials-12-03973-s001.zip › nanomaterials-1995717-supplementary.pdf]

Supplementary Materials

Rational Design of Fluorinated Phthalonitrile/Hollow Glass Microsphere Composite with Low Dielectric Constant and Excellent Heat Resistance for Microelectronic Packaging

Minjie Wu [1], Wenshuang Han [1], Chun Zhang [1], Shuo Zhang [1], Xinyang Zhang [1], Xinggang Chen [2], Kimiyoshi Naito [3], Xiaoyan Yu [1,*] and Qingxin Zhang [1,4,*]

[1] Hebei Key Laboratory of Functional Polymers, School of Chemical Engineering and Technology, Hebei University of Technology, Tianjin 300401, China
[2] School of Materials Science and Engineering, North China University of Science and Technology, Tangshan 063210, China
[3] National Institute for Materials Science (NIMS), Hybrid Materials Unit, Composite Materials Group, 1-2-1 Sengen, Tsukuba 305-0047, Japan
[4] Tianjin Key Laboratory of Materials Laminating Fabrication and Interface Control Technology, Hebei University of Technology, Tianjin 300401, China
* Correspondence: yuxycnn@163.com (X.Y.); zhqxcn@163.com (Q.Z.)

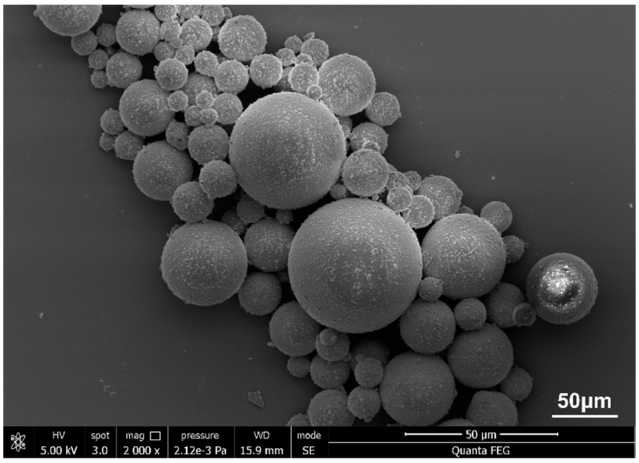

Figure S1. SEM microsgraph of the original HGM

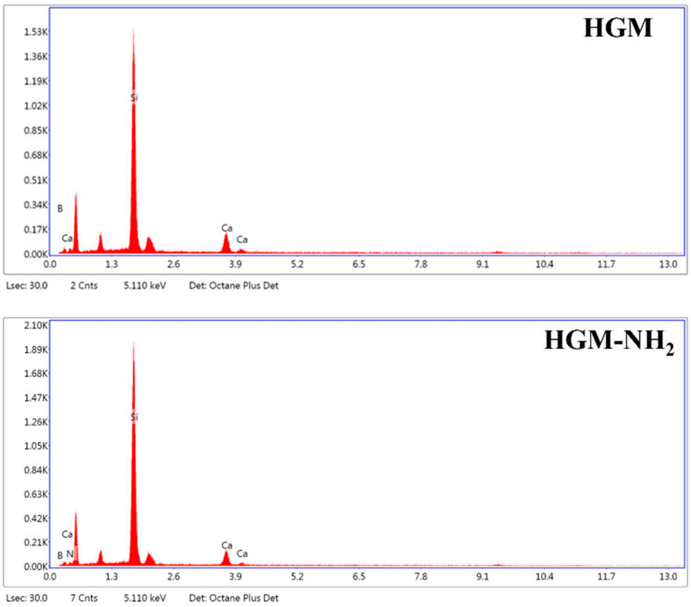

Figure S2. EDS spectra of HGM and HGM-NH_2

Table S1. HGM and HGM-NH_2 atomic content determined by EDS spot scanning

Element	Atomic %	
	HGM	HGM-NH_2
B	33.04	28.00
Si	55.98	62.28
Ca	10.98	9.56
